# Painful to Discuss: The Intersection of Chronic Pain, Mental Health, and Analgesic Use among People with HIV

**DOI:** 10.33696/aids.5.046

**Published:** 2023

**Authors:** Sara D. PULLEN, Maria Anjanette NUÑEZ, Sydney BENNETT, Wellsley BROWN, Catherine CRONIN, Maya FLEISCHER, Abigail MALCOLM, Vijay RAMESH, Kayla SPENCER

**Affiliations:** 1 Emory University School of Medicine, Department of Rehabilitation Medicine, Atlanta, GA 30322, USA; 2 Emory Center for AIDS Research, Atlanta, GA 30322, USA

**Keywords:** HIV, AIDS, Chronic pain, Trauma, Analgesics, Mental health

## Abstract

**Objective:**

This retrospective chart review study aims to identify patients in an HIV clinical setting in an area of high HIV prevalence in Atlanta, Georgia, USA who have chronic pain, analgesic prescriptions, and/or mental health diagnoses.

**Design:**

People living with HIV (PLWH) are at higher risk for experiencing trauma, mental health conditions, and chronic pain than their HIV-negative counterparts. This study was designed to evaluate the intersection of these factors within an urban HIV clinic.

**Methods:**

Retrospective chart review study.

**Results:**

Of the adult patients enrolled at an HIV clinic in Atlanta, Georgia USA between 2011–2022 (n=15,970), 93.7% were prescribed analgesics, 40.5% had documented pain diagnoses, and 23.5% had documented mental health diagnoses. Additionally, 14.3% of all enrolled patients had all three factors concurrently.

**Conclusions:**

The complexity of HIV, chronic pain, mental health challenges, and analgesic use demand a patient-centered, collaborative approach including a multidisciplinary care team. Seeing persistent pain among PLWH with a trauma-informed approach to care within the lens of co-occurring mental health diagnoses will allow us to better understand, treat, and sustain patients in life-saving HIV care.

## Introduction

Chronic pain has been widely defined as lasting beyond normal tissue healing time, delineated at three months since time of pain onset (International Association for the Study of Chronic Pain) [1,2]. Accepted treatment for chronic pain pairs pharmacological and non-pharmacological, multifaceted pain management that considers the biopsychosocial nature of pain [3,4]. Pharmacological treatment guidelines are based on individual patient circumstance and past medical history [5]. The World Health Organization (WHO)’s Analgesic Ladder has been widely accepted as a guided approach to use of analgesics for chronic pain [6]. Non-pharmacological management of pain is noted to encompass behavioral, cognitive, integrative, and physical therapies to encourage tissue healing while restoring functional mobility [7]. Notably, exercise appears to produce analgesic effects for chronic pain when appropriately dosed and should be considered as a key non-pharmacologic component of pain management [8]. Physical activity and cognitive behavioral therapy have been shown to help modulate chronic pain as well as improve overall health and quality of life in individuals [9,10].

Chronic pain is one of the most frequently reported symptoms in PWH and can have drastic impacts on patient quality of life [12]. One analysis of PWH over a three-year period found that 55% of subjects reported experiencing pain, and among those subjects, 82% of subjects described their pain as severe or very severe [13]. Other investigations have found that chronic pain affects patient retention, and is possibly related higher rates of virologic failure [14]. Even if HIV is virologically controlled by anti-retroviral therapy (ART), chronic pain often persists and has been found to have a multifactorial etiology [12]. As with other chronic pain, women are more likely to have chronic HIV-related pain and are at a higher risk for under-treatment of their pain [12]. Persistent pain frequently has drastic impacts for PWH, as it often has complex relationships with mental health, care retention, and adherence to treatment [14–16]. A potential weakness in these analyses is that they rely on self-reported pain, which may be inconsistent and impacted by a variety of factors [17].

Long-term analgesic use for PWH is more complex due to high prevalence of comorbid mood disorders, substance abuse histories, hyperalgesia, and drug interactions [18,19]. Persons living with HIV on ART may be at higher risk for potential drug-drug interactions (pDDIs) due to polypharmacy, including between over-the-counter and prescription analgesics and ART [17,20–22]. HIV enters the brain within 4–8 days after initial infection and establishes CNS reservoirs, even in the context of ART [11]. Opioid analgesics impact immune cells via opioid receptor binding and cross desensitization with chemokine receptors. Therefore, opioid use in PWH can be controversial within the complex relationship between opioids and HIV in the CNS [11]. Recent studies found that following a course of physical therapy, PWH reported decreased pain and decreased usage of NSAIDs, acetaminophen, and neuropathic pain medications; however, opioid usage remained the same [15,23]. This can be attributed to the fact that weaning of opioids is a more complex issue correlated with prior substance abuse and nuances in mental health, thus requiring more time and intervention to cease its use [24]. Consequently, physical therapy should be considered an effective means of treating pain in this patient population [15].

Taking a biopsychosocial lens to chronic pain requires a complementary, diverse framework via multiple health care disciplines. Current protocols have shown that an integrative multidisciplinary approach including physical therapy and behavioral health interventions yields benefits beyond physiological pain management including decreased reliance on analgesics and reduction in unnecessary medical intervention [4].

### Trauma and HIV

Trauma is defined by the Substance Abuse and Mental Health Services Administration (SAMHSA) as “an event experienced by an individual physically or emotional harmful and/or life-threatening and that has lasting adverse effects on an individual’s functioning and mental, physical, social, emotional, or spiritual well-being” [33]. It is estimated that 51–81% of all adults in high-income counties, such as the US, have experienced a minimum of one traumatic event, with a great portion of those same individuals reporting experiencing multiple traumatic events throughout their lifetime [34]. There is an even higher prevalence of trauma among PWH: one study estimates that 68%–98% of women, 68%–77% of men, and 93% of transgender people with HIV within the United states have experienced some sort of trauma, with 30% of all HIV infected individuals experiencing physical and/or sexual abuse before the age of 13 [34]. Post-traumatic stress disorder (PTSD) among PWH is as high as 74% compared to only 8% among the general US population [29]. Once trauma is experienced, it can affect individuals well beyond the initial incident, resulting in lasting emotional and physical consequences. Those who have experienced trauma are also more likely to experience a higher incidence of pain, more pain conditions, and increased severity of pain in comparison to their HIV-negative counterparts [34,35]. Up to 50% of PWH with chronic pain report having PTSD in comparison to only 6–12% of their HIV negative counterparts [35]. While this likely has a multifaceted etiology, this may be at least partially attributed to lower thresholds for physiologic pain found in those with both chronic pain and trauma exposure [35].

A systematic review of mental health interventions for PWH in low and middle-income countries reports that HIV and mental health are bidirectional, indicating that living with HIV increases the risk of experiencing a mental health condition, but having a mental health condition also increases one’s risk of contracting HIV [32].

Effectiveness of treatment for PWH has made significant strides in the past three decades with early and consistent care. Early initiation of ART has been confirmed to reduce the risk of developing HIV opportunistic illnesses, increase life expectancy, improve access to supportive services, and improve overall quality of life [36,37]. However, there are a multitude of system-related and patient-related barriers that affect the ability to access and receive proper treatment for individuals living with HIV. System-related barriers can include insurance or financial constraints, lack of access to HIV specialty providers and privacy concerns [37]. Common patient-related barriers can include mental health and substance use disorders, limited social support, avoidance and denial of status, and HIV stigma [37]. HIV-related stigma continues to be highly correlated with decreased consistency with ART, limited access to medical care and decreased quality of life in PWH [36,38]. Eliminating system-related and patient-related barriers is essential to providing effective and necessary treatment to individuals living with HIV to successfully improve their overall quality of life.

People with HIV are at higher risk for experiencing trauma, mental health conditions, and chronic pain. Taking a biopsychosocial lens to chronic pain requires a complementary, diverse framework via multiple health care disciplines. Current protocols have shown that an integrative multidisciplinary approach including physical therapy and behavioral health interventions yields benefits beyond physiological pain management including decreased reliance on analgesics and reduction in unnecessary medical intervention [4]. This study aims to identify patients in an HIV clinical setting with documented analgesic use, mental health diagnoses, and/or chronic pain via retrospective chart review from a study site in Atlanta, Georgia, USA.

Metropolitan Atlanta was an ideal location for this study, as the burden of HIV disease is high: Atlanta is ranked 2^nd^ in the nation for total number of new diagnoses of HIV for the year 2018, which is the most recent data available at time of authorship [39]. The Ponce de Leon Center has over 6,200 enrolled HIV-positive patients, with approximately 90% of these patients identifying with under-represented minority groups. Over 82% of enrolled patients live below the federal poverty level (with annual income below $27,000); 42% are uninsured and 26% receive Medicaid. More than 70% of PLHIV who live in Atlanta reside within two miles of the Ponce Clinic, in an area recognized as a spatial clustering of the Atlanta HIV epidemic [40]. This study population in Atlanta, Georgia is a representative sample of PWH in the United States. In the United States, 92% of PWH identify as under-represented minorities compared to 90% in Atlanta [39,40]. In the United States, 75% of PHW live below the federal poverty level compared to 82% in Atlanta [40,41].

The complexity of HIV, mental health challenges, and chronic pain demand a multi-layered approach to understanding, treating, and improving quality of life for PWH. Addressing persistent pain among PWH with a trauma-informed approach to care within the lens of co-occurring mental health diagnoses will allow us to better understand, treat, and sustain patients in life-saving HIV care.

## Methods

Inclusion criteria for this study included adult HIV-positive individuals older than 18 years of age, enrolled at the Ponce de Leon Center in Atlanta, Georgia, the USA with a listed chronic pain diagnosis, and/or mental health diagnosis, and/or analgesic since the inception of the HIV Data Registry in 2011 (n = 16,798).

This study was deemed exempt from Institutional Review Board review from the authors’ home institution, as it only utilized secondary data from de-identified patient charts. Data was collected from The Emory Center for AIDS Research (CFAR) HIV Disease Registry application at Grady Health System. The Registry contains data abstracted from the electronic medical records (EMR) of patients who received medical treatment at the Ponce de Leon Center IDP since 2011. The CFAR application is self-contained, served through a stand-alone HIPAA compliant Oracle-based connection hosted on Emory servers, with no connection or interface to any patient records system, desktop-installed component, or other external systems. It contains individual-level patient data including demographics, encounter information, diagnostic codes, and laboratory data on over HIV-positive persons. Data security is maintained via secured two-factor authentication with VPN access only. Data was collected and organized by one designated data analyst from the HIV Disease Registry and shared with the Primary Investigator via a password-protected document emailed securely within the home institution’s server.

Data was collected for two periods of time. The first timeframe (Sample 1) spanned from the inception of data monitoring by the Data Registry in 2011 until 2022. The second (Sample 2) spanned a two-year time frame from 2020 to 2022. The Sample 2 timeframe was chosen to examine whether these percentages changed over time and if they were affected significantly by the COVID-19 pandemic years. Analgesic use was considered present if listed twice over a period of at least 90 days from the lists of classes on the NIH National Library of Medicine Rx Classes including: analgesics [42], non-steroidal anti-inflammatory agents [43], opioid analgesics [44], and opioids in combination with other non-opioid analgesics [45], in addition to Pregabalin, Duloxetine, and Gabapentin. Mental health diagnoses included the following conditions, identified using ICD codes: depression, anxiety, post-traumatic stress disorder, psychological trauma, and physical and sexual abuse (Appendix 1). Pain diagnoses included the ICD-10 codes for generalized pain, chronic pain, neck pain, pain in joint, shoulder pain, ankle pain, knee pain, hip pain, lumbago, low back pain, thoracic pain, pain in limb, unspecified idiopathic peripheral neuropathy, headache, osteoarthritis, sciatica, and neuropathy (Appendix 2). Quantitative analysis was performed using Microsoft Excel software. Data collected from the registry was inserted, organized, and numerically coded through Excel (Appendix 3) [15].

## Results

Data was collected examining the intersection of chronic pain, mental health diagnoses, and analgesic use among adults (over age 18) with HIV enrolled at the Grady Ponce de Leon Center from 2011–2022 (n= 15,970).

**Figure 1.**
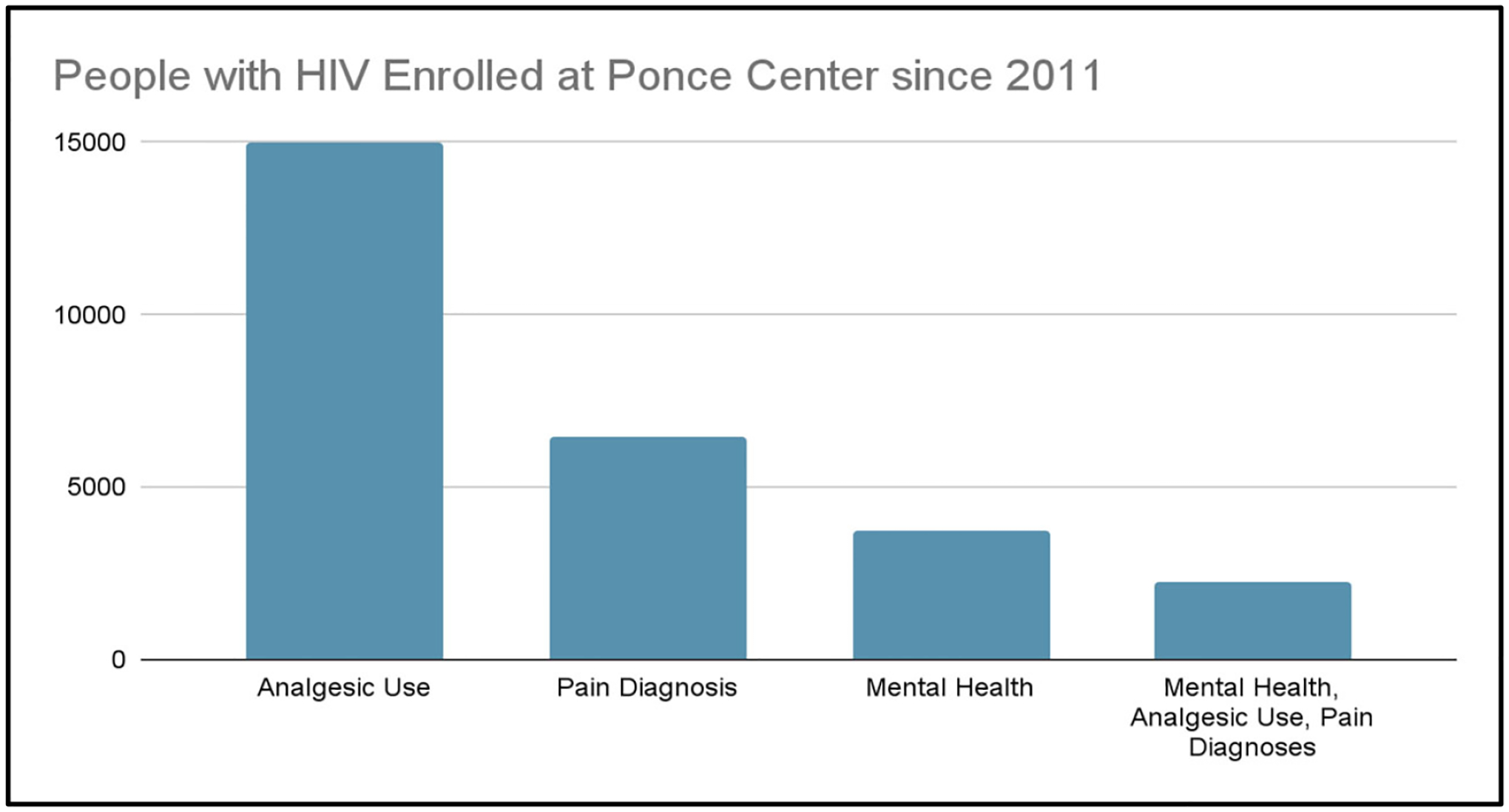
People with HIV Enrolled at Ponce Center, 2011–2022.

**Figure 2.**
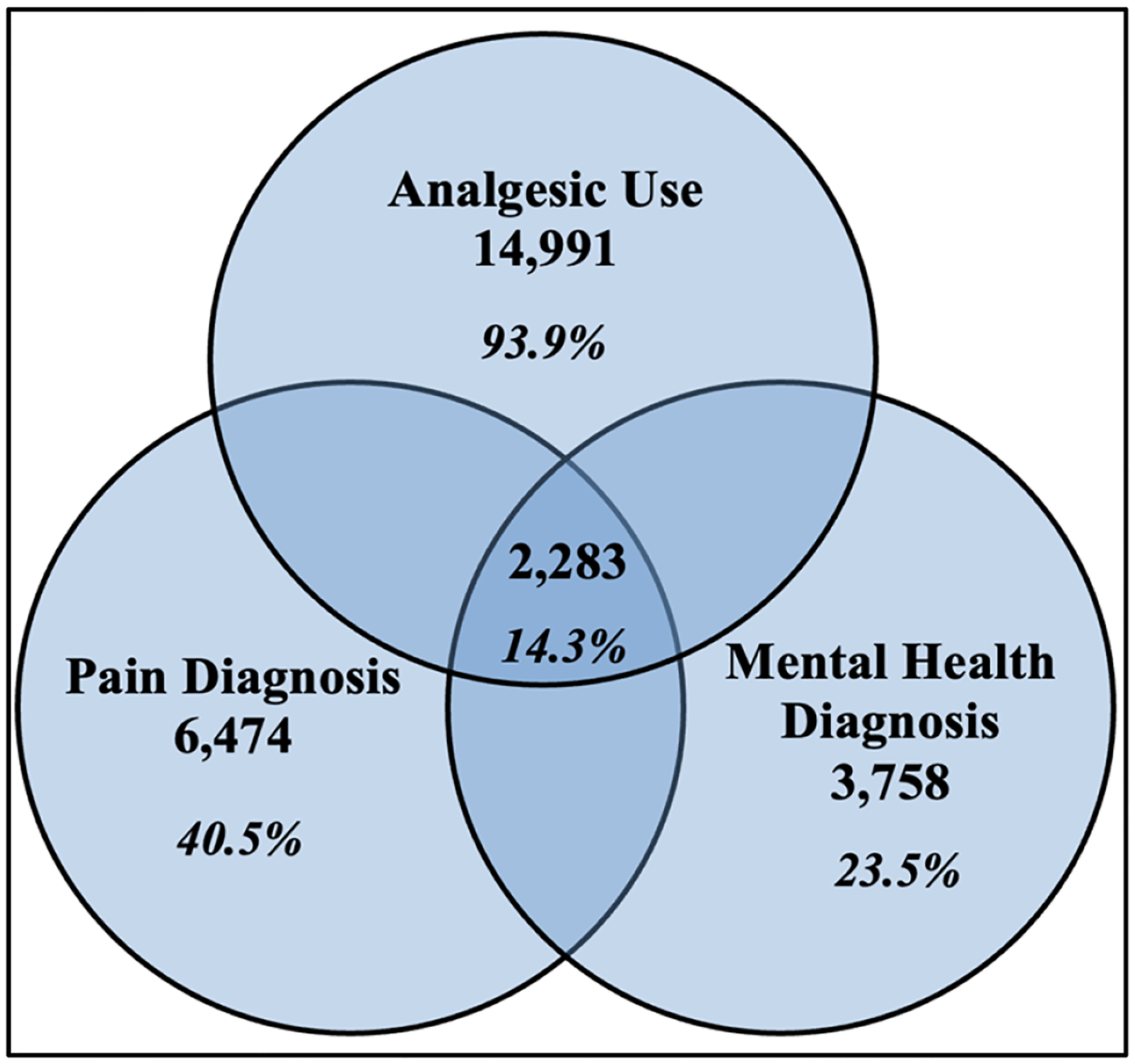
Pain, Analgesics, and Mental Health Diagnoses, 2011–2022.

Figure 1 details People with HIV enrolled at the Grady Ponce de Leon Center 2011–2022. Figure 2 shows Pain, Analgesics and Mental Health Diagnoses, 2011–2022.

Of the adult patients enrolled at the Grady Ponce Center with HIV since 2011 (Sample 1, n=15,970), 93.7% of patients (n = 14,991) were prescribed analgesics, 40.5% of patients (n = 6,474) had documented pain diagnoses, and 23.5% of patients (n = 3,758) had documented mental health diagnoses. Additionally, 14.3% of patients (n = 2283) had documented analgesic use, pain diagnoses, and mental health diagnoses together (Figure 2).

Sample 2 data was collected examining the intersection of chronic pain, mental health diagnoses, and analgesic use among adults (over age 18) with HIV enrolled at the Grady Infectious Disease clinic from 2020–2022 (n= 6400).

**Figure 3.**
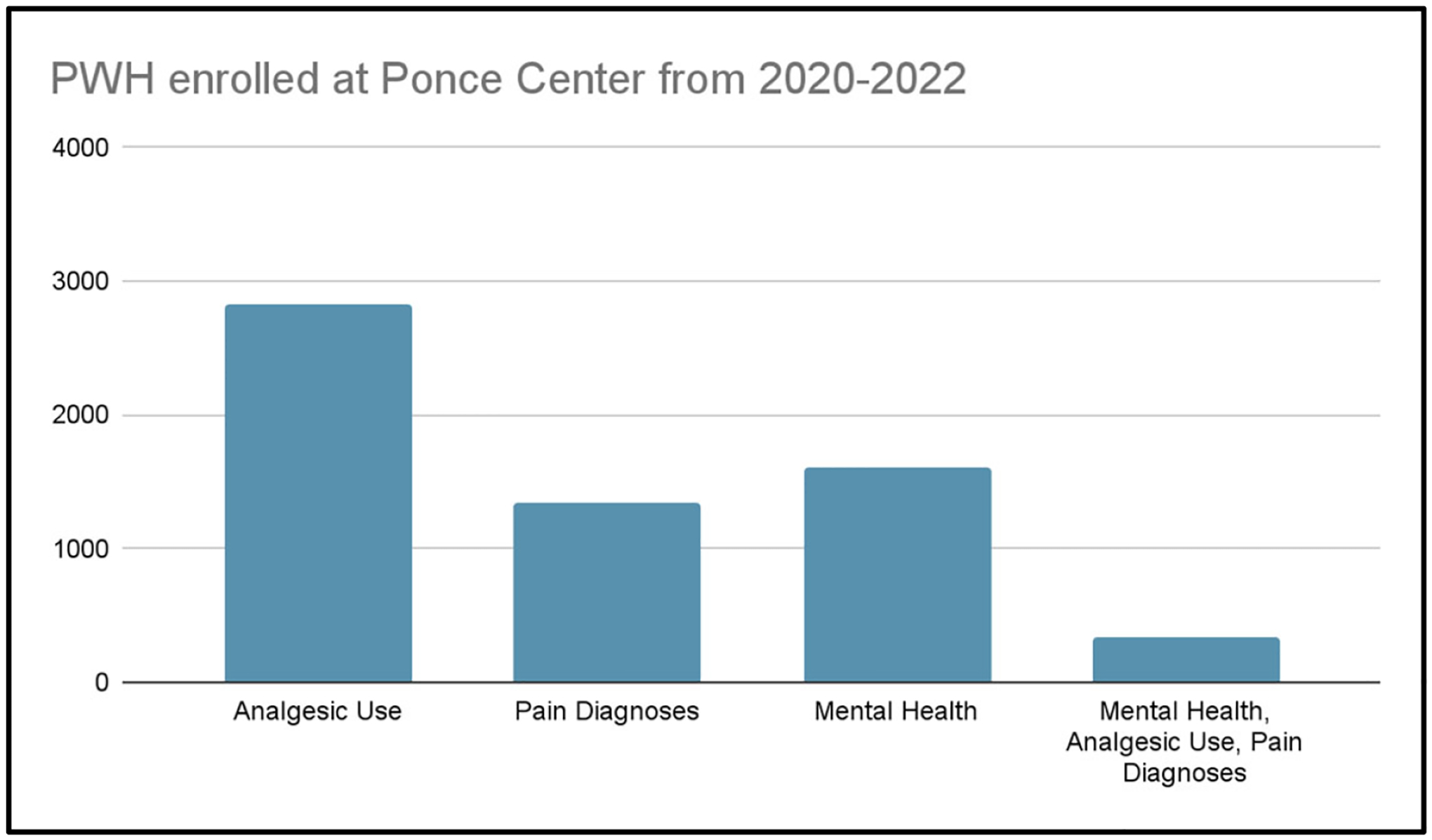
Enrolled patients at Ponce Center, 2020–2022.

**Figure 4.**
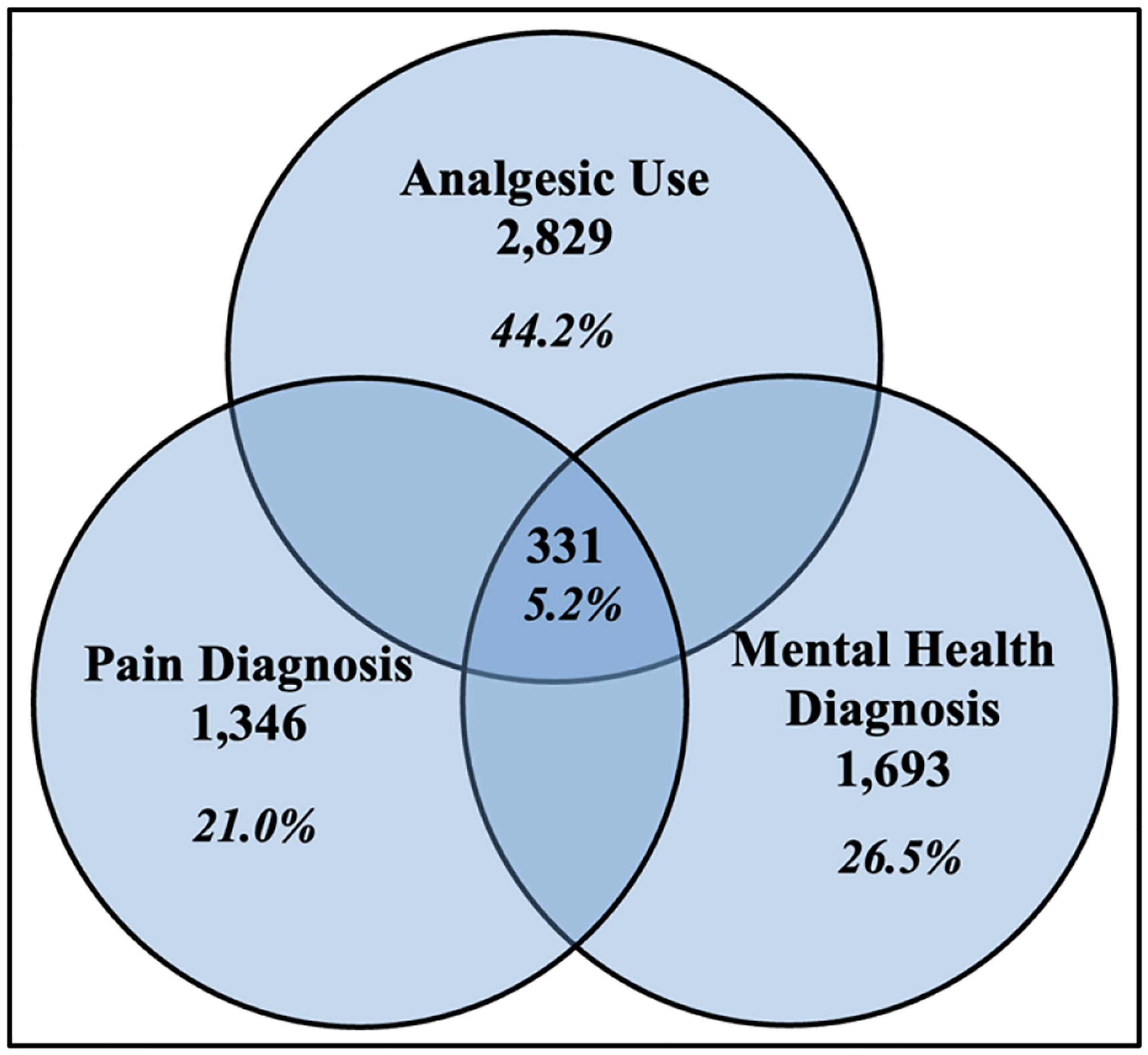
Pain, Analgesic, and Mental Health Diagnoses, 2020–22.

Figure 3 shows enrolled patients at Ponce Center from 2020–2022. Figure 4 shows Pain, Analgesic and Mental Health Diagnoses, 2020–22.

From Figures 3 and 4: Of the adult patients enrolled at the Grady Ponce Center with HIV from 2020 – 2022 (n=6,400), 44.2% of patients (n = 2,829) were prescribed analgesics, 21.0% of patients (n=1,346) had documented pain diagnoses, and 26.5% of patients (n = 1,693) had documented mental health diagnoses. Additionally, 5.2% of patients (n = 331) had documented concurrent analgesic use, pain diagnoses, and mental health diagnoses.

The percentages of enrolled patients with all documented factors decreased during the years of 2020–2022 (Table 1):

## Discussion

People with HIV (PWH) are at higher risk for experiencing trauma, mental health conditions, and chronic pain than their HIV-negative counterparts. These overlapping complexities demand a multi-layered approach to understanding, treating, and improving quality of life for PWH. In our study of adults enrolled at an urban HIV clinic in an area of high HIV prevalence, data revealed that across 11 years from 2011 through 2022 (n=15,970), 14.3% experienced an intersection of all three variables of chronic pain, analgesic use, and mental health challenges, including trauma. With the same patient population across a 2-year period from 2020 through 2022 (n=6,400), 5.17% (n=331) experienced an intersection of all three variables.

Currently there is limited research about how these three variables - all more prevalent among PWH - intersect and affect individuals within the complexity of HIV. While the effectiveness of treatment for PWH and the provision of earlier and more consistent care have been greatly enhanced in the last few decades, there are still great strides to be made to provide optimal care. System-related and patient-related barriers often prevent the proper treatment for those living with HIV. Considering the majority, nearly 100%, of those with an HIV diagnosis at the Grady Ponce Center in Atlanta are prescribed analgesics while only 40.5% had a documented pain diagnosis, we must consider all the variables included in a patient’s experience of and treatment for pain.

The percentages of enrolled patients with all documented factors (pain, analgesic use, and mental health diagnoses) decreased during the years of 2020–2022, which were the peak years of the COVID-19 pandemic. This may be due to the fact that clinic visits decreased during this time overall, and/or that patients’ visits were shorter and therefore focused on HIV status more than concurrent diagnoses.

The widely accepted best practice for treating individuals with chronic pain utilizes a biopsychosocial framework to address the multiple facets that inform a person’s pain experience. It is significant that our data shows relatively low pain diagnoses when compared to prescribed analgesics and highlights the need for a multi-faceted approach to pain and close documentation of pain within an HIV clinical visit. If pain is being underdiagnosed while analgesic prescription remains high, it may foster worse outcomes, drug interactions or medication dependence if the analgesics being prescribed have habit-forming qualities. Measuring any analgesic use over a period of 11 years may not be correlated with chronic pain, as patients often utilize analgesics for acute and/or temporary pain. By looking at the intersection of chronic pain *and* analgesic use, we hoped to capture analgesics being used in a long-term context.

The higher prevalence of chronic pain and trauma among PWH is well documented, as is the connection of trauma and chronic pain in non-HIV specific populations. The event of being diagnosed with HIV can be traumatic in itself due to the social stigma surrounding the disease and the complications that can arise from the disease progression. Given this, it is notable that only about one quarter of PWH enrolled at the Grady Ponce Center have diagnoses of mental health disorders. Ultimately, best practices for treatment of pain in PWH require efficient identification of accompanying individual health conditions including mental health.

The complexity of chronic pain is best addressed with a holistic, interdisciplinary framework in all clinical settings, including HIV care. Implementing a sustainable, collaborative approach to persistent pain with a patient-centered team can improve clinical communication and improve patient outcomes [46].

This study presented several limitations. One limitation is that only prescribed analgesics were accounted for in the data. Any analgesics obtained over the counter, street drugs (heroin, fentanyl), and usage of prescription-grade analgesics not prescribed to the patient (i.e. taking a friend or family member’s medication or purchasing them in a private transaction). A second limitation is regarding the number of individuals with a mental health diagnosis. Patients may not have received an official ICD-10 code if the mental health condition was solely acknowledged in the subjective portion of documentation and therefore not included as a separate diagnosis during the visit. Finally, another limitation is that this sample size was only from one site - the Grady Ponce De Leon Center in Atlanta, GA. Physical therapy (PT) is a widely accepted means of managing chronic pain and there is a growing body of research to support PT for chronic pain among PWH. However, there is currently only one HIV treating physical therapist working at the Ponce De Leon Center. While the clinic utilizes a collaborative healthcare approach, there is limited availability for patients to receive PT treatments.

## Conclusion

This study provides insight within a representative sample to better understand the intersection between pain, analgesic use, and mental health among PWH. However, there are gaps in the literature about how these factors interact with one another, specifically in those living with HIV. This study informs a direction for better understanding these relationships to provide a multi-disciplinary and integrative approach to care for PWH to improve overall quality of life. With these findings, there is an intent to create a screening tool to better identify candidates for physical therapy and mental health services in order to provide more successful and holistic approaches to treating individuals with HIV experiencing chronic pain and mental health challenges.

Currently, there is limited provision of holistic and interdisciplinary care for chronic pain in all settings - including HIV - which has been shown to be the most successful when treating individuals with complex pain. It is imperative to properly identify patients that are candidates for more interdisciplinary care, including physical therapy and mental health resources, in addition to primary care. Future research should aim to create a screening tool to better identify PWH that would benefit from holistic and interdisciplinary care as a means of treating chronic pain and improving their overall quality of life.

## Supplementary Material

JAHT-23-046_Supplementary_File

## Figures and Tables

**Table 1.** Change in Diagnosis Prevalence, Samples 1 and 2.

	2011–2022	2020–2022	% Change
**Analgesic use only**	14,991 (93.9%)	2,829 (44.2%)	↓ 52.9%
**Pain diagnosis only**	6,474 (40.5%)	1,346 (21%)	↓ 48.1%
**Mental health diagnosis only**	3,758 (23.5%)	1,693 (26.5%)	↑ 12.8%
**Intersection of all three**	2,283 (14.3%)	331 (5.17%)	↓ 63.9%

## References

[1] Hylands-White N, Duarte RV, Raphael JH. An overview of treatment approaches for chronic pain management. Rheumatology International. 2017 Jan;37:29–42. 27107994 10.1007/s00296-016-3481-8

[2] Raja SN, Carr DB, Cohen M, Finnerup NB, Flor H, Gibson S, Keefe FJ, The revised International Association for the Study of Pain definition of pain: concepts, challenges, and compromises. Pain. 2020 Sep 1;161(9):1976–82. 32694387 10.1097/j.pain.0000000000001939 PMC7680716

[3] Sarzi-Puttini P, Vellucci R, Zuccaro SM, Cherubino P, Labianca R, Fornasari D. The appropriate treatment of chronic pain. Clinical Drug Investigation. 2012 Feb;32:21–33. 10.2165/11630050-000000000-00000 22356221

[4] Danilov A, Danilov A, Barulin A, Kurushina O, Latysheva N. Interdisciplinary approach to chronic pain management. Postgraduate Medicine. 2020 Nov 16;132(sup3):5–9. 32298161 10.1080/00325481.2020.1757305

[5] Park HJ, Moon DE. Pharmacologic management of chronic pain. The Korean Journal of Pain. 2010 Jun 30;23(2):99–108. 20556211 10.3344/kjp.2010.23.2.99 PMC2886242

[6] Anekar AA, Cascella M. WHO Analgesic Ladder. In: StatPearls. Treasure Island (FL): StatPearls Publishing; November 15, 2022. 32119322

[7] Chang KL, Fillingim R, Hurley RW, Schmidt S. Chronic pain management: nonpharmacological therapies for chronic pain. FP Essentials. 2015 May 1;432:21–6. 25970869

[8] Courtney CA, Fernández-de-Las-Peñas C, Bond S. Mechanisms of chronic pain–key considerations for appropriate physical therapy management. Journal of Manual & Manipulative Therapy. 2017 May 27;25(3):118–27. 28694674 10.1080/10669817.2017.1300397 PMC5498792

[9] Wang H, Hu F, Lyu X, Jia H, Wang B, Liu F, Kinesiophobia could affect shoulder function after repair of rotator cuff tears. BMC Musculoskeletal Disorders. 2022 Dec;23(1):714. 35883122 10.1186/s12891-022-05679-x PMC9316366

[10] Pacheco-da-Costa S, Soto-Vidal C, Calvo-Fuente V, Yuste-Sánchez MJ, Sánchez-Sánchez B, Asúnsolo-del-Barco Á. Evaluation of Physical Therapy Interventions for Improving Musculoskeletal Pain and Quality of Life in Older Adults. International Journal of Environmental Research and Public Health. 2022 Jun 8;19(12):7038. 35742284 10.3390/ijerph19127038 PMC9223092

[11] Murphy A, Barbaro J, Martínez-Aguado P, Chilunda V, Jaureguiberry-Bravo M, Berman JW. The effects of opioids on HIV neuropathogenesis. Frontiers in Immunology. 2019 Oct 18;10:2445. 31681322 10.3389/fimmu.2019.02445 PMC6813247

[12] Addis DR, DeBerry JJ, Aggarwal S. Chronic pain in HIV. Molecular Pain. 2020 May;16:1744806920927276. 10.1177/1744806920927276 PMC7252379 32450765

[13] García-Dasí M, Pérez-Alenda S, Carrasco JJ, Marques-Sule E, Aguilar-Rodríguez M, Moreno-Segura N, Effects of a non-pharmacological approach for chronic pain management in patients with haemophilia: efficacy of cognitive-behavioural therapy associated with physiotherapy. Haemophilia. 2021 May;27(3):e357–67. 33650767 10.1111/hae.14284

[14] Merlin JS, Long D, Becker WC, Cachay ER, Christopoulos KA, Claborn K, Brief report: the association of chronic pain and long-term opioid therapy with HIV treatment outcomes. Journal of Acquired Immune Deficiency Syndromes. 2018 Sep 1;79(1):77–82. 29771793 10.1097/QAI.0000000000001741 PMC6092197

[15] Pullen SD, Del Rio C, Brandon D, Colonna A, Denton M, Ina M, Associations between chronic pain, analgesic use and physical therapy among adults living with HIV in Atlanta, Georgia: a retrospective cohort study. AIDS Care. 2020 Jan 2;32(1):65–71. 10.1080/09540121.2019.1661950 PMC8261858 31529994

[16] Merlin JS, Westfall AO, Raper JL, Zinski A, Norton WE, Willig JH, Pain, mood, and substance abuse in HIV: implications for clinic visit utilization, ART adherence, and virologic failure. Journal of Acquired Immune Deficiency Syndromes. 2012 Oct 10;61(2):164–70. 22766967 10.1097/QAI.0b013e3182662215 PMC3459261

[17] Parker R, Stein DJ, Jelsma J. Pain in people living with HIV/AIDS: a systematic review. Journal of the International AIDS Society. 2014 Jan;17(1):18719. 24560338 10.7448/IAS.17.1.18719 PMC3929991

[18] Surratt HL, Kurtz SP, Levi-Minzi MA, Cicero TJ, Tsuyuki K, O’Grady CL. Pain treatment and antiretroviral medication adherence among vulnerable HIV-positive patients. AIDS Patient Care and STDs. 2015 Apr 1;29(4):186–92. 24984142 10.1089/apc.2014.0104 PMC4378712

[19] Tsao JC, Stein JA, Dobalian A. Pain, problem drug use history, and aberrant analgesic use behaviors in persons living with HIV. PAIN^®^. 2007 Dec 15;133(1–3):128–37. 17449182 10.1016/j.pain.2007.03.016 PMC2173909

[20] Edelman EJ, Rentsch CT, Justice AC. Polypharmacy in HIV: recent insights and future directions. Current Opinion in HIV and AIDS. 2020 Mar;15(2):126. 31833963 10.1097/COH.0000000000000608 PMC7543953

[21] Johnston JP, Heavner MS, Liu M, Casal GL, Akgün KM. The Prevalence of Drug–Drug Interactions with Antiretroviral Therapy in Human Immunodeficiency Virus–Infected Patients in the Intensive Care Unit. Journal of Pharmacy Practice. 2023 Apr;36(2):322–8. 34587846 10.1177/08971900211035262

[22] Passik SD, Kirsh KL, Donaghy KB, Portenoy RK. Pain and aberrant drug-related behaviors in medically ill patients with and without histories of substance abuse. The Clinical Journal of Pain. 2006 Feb 1;22(2):173–81. 16428952 10.1097/01.ajp.0000161525.48245.aa

[23] Pullen S. Physical therapy as non-pharmacological chronic pain management of adults living with HIV: self-reported pain scores and analgesic use. HIV/AIDS-Research and Palliative Care. 2017 Sep 18:177–82. 10.2147/HIV.S141903 PMC5609779 29075140

[24] Pullen SD, Del Rio C, Brandon D, Colonna A, Denton M, Ina M, An innovative physical therapy intervention for chronic pain management and opioid reduction among people living with HIV. BioResearch Open Access. 2020 Dec 1;9(1):279–85. 33376634 10.1089/biores.2020.0006 PMC7757684

[25] Mental disorders. World Health Organization. https://www.who.int/news-room/fact-sheets/detail/mental-disorders. June 8, 2022. Accessed December 13, 2022.

[26] Mental Health Treatments. Mental Health America. https://mhanational.org/mental-health-treatments. (n.d.). Accessed November 2, 2022.

[27] Mental Health Medications. National Institute of Mental Health (NIMH). https://www.nimh.nih.gov/health/topics/mental-health-medications. (n.d.). Accessed November 2, 2022.

[28] Schomerus G, Stolzenburg S, Freitag S, Speerforck S, Janowitz D, Evans-Lacko S, Stigma as a barrier to recognizing personal mental illness and seeking help: a prospective study among untreated persons with mental illness. European Archives of Psychiatry and Clinical Neuroscience. 2019 Jun 1;269:469–79. 29679153 10.1007/s00406-018-0896-0

[29] Remien RH, Stirratt MJ, Nguyen N, Robbins RN, Pala AN, Mellins CA. Mental health and HIV/AIDS: the need for an integrated response. AIDS (London, England). 2019 Jul 7;33(9):1411–20. 30950883 10.1097/QAD.0000000000002227 PMC6635049

[30] Bing EG, Burnam MA, Longshore D, Fleishman JA, Sherbourne CD, London AS, Psychiatric disorders and drug use among human immunodeficiency virus–infected adults in the United States. Archives of General Psychiatry. 2001 Aug 1;58(8):721–8. 11483137 10.1001/archpsyc.58.8.721

[31] Substance Abuse and Mental Health Services Administration. Behavioral health trends in the United States: results from the 2014 National Survey on Drug Use and Health, 2015. HHS Publication No. SMA 15–4927, NSDUH Series H-50.

[32] Nakimuli-Mpungu E, Musisi S, Smith CM, Von Isenburg M, Akimana B, Shakarishvili A, Nachega JB, Mental health interventions for persons living with HIV in low-and middle-income countries: a systematic review. Journal of the International AIDS Society. 2021 Jun;24:e25722. 34164926 10.1002/jia2.25722 PMC8222847

[33] Nadal KL, Erazo T, King R. Challenging definitions of psychological trauma: Connecting racial microaggressions and traumatic stress. Journal for Social Action in Counseling & Psychology. 2019 Dec 12;11(2):2–16.

[34] Sales JM, Swartzendruber A, Phillips AL. Trauma-informed HIV prevention and treatment. Current HIV/AIDS Reports. 2016 Dec;13:374–82. 27704251 10.1007/s11904-016-0337-5 PMC5107145

[35] Salahuddin D, Conti T. Trauma and behavioral health care for patients with chronic pain. Primary Care: Clinics in Office Practice. 2022 Sep 1;49(3):415–23. 36153083 10.1016/j.pop.2022.04.001

[36] Holzemer WL, Human S, Arudo J, Rosa ME, Hamilton MJ, Corless I, Exploring HIV stigma and quality of life for persons living with HIV infection. Journal of the Association of Nurses in AIDS Care. 2009 May 1;20(3):161–8. 10.1016/j.jana.2009.02.002 19427593

[37] Mgbere O, Khuwaja S, Bell TK, Rodriguez-Barradas MC, Arafat R, Essien EJ, System and patient barriers to care among people living with HIV/AIDS in Houston/Harris County, Texas: HIV medical care providers’ perspectives. Journal of the International Association of Providers of AIDS Care (JIAPAC). 2015 Nov;14(6):505–15. 24943655 10.1177/2325957414539045

[38] Turan B, Hatcher AM, Weiser SD, Johnson MO, Rice WS, Turan JM. Framing mechanisms linking HIV-related stigma, adherence to treatment, and health outcomes. American Journal of Public Health. 2017 Jun;107(6):863–9. 28426316 10.2105/AJPH.2017.303744 PMC5425866

[39] Centers for Disease Control and Prevention. Diagnoses of HIV infection, 2018, and persons living with diagnosed HIV infection (prevalence), year-end 2017, by metropolitan statistical area of residence. 2018. Retrieved from https://www.cdc.gov/hiv/pdf/library/reports/surveillance/cdc-hiv-surveillance-report-2018-preliminary-vol-30.pdf. Accessed March 27, 2023.

[40] Centers for Disease Control and Prevention. 2018. HIV in the United States: At a glance. Retrieved from https://www.cdc.gov/hiv/pdf/library/reports/surveillance/cdc-hiv-surveillance-report-2016-vol-28.pdf. Accessed March 21, 2023.

[41] Denning P, DiNenno E. Communities in crisis: is there a generalized HIV epidemic in impoverished urban areas of the United States. In: XVIII International AIDS Conference. 2010 Jul 18.

[42] Rxclass: analgesics. U.S. National Library of Medicine. https://mor.nlm.nih.gov/RxClass/search?query=N02%7CATC1-4&searchBy=class&sourceIds=&drugSources=atc1-4%7Catc%2Cepc%7Cfdaspl%2Cmeshpa%7Cmesh%2Cdisease%7Cndfrt%2Cchem%7Cfdaspl%2Cmoa%7Cfdaspl%2Cpe%7Cfdaspl%2Cpk%7Cndfrt%2Cva%7Cndfrt%2Ctc%7Cfmtsme%2Cdispos%7Csnomedct%2Cstruct%7Csnomedct%2Ctherap%7Csnomedct. Accessed March 14, 2023.

[43] Rxclass: NSAIDs. U.S. National Library of Medicine. https://mor.nlm.nih.gov/RxClass/search?query=Anti-Inflammatory+Agents%2C+Non-Steroidal%7CMESHPA&searchBy=class&sourceIds=&drugSources=atc1-4%7Catc%2Cepc%7Cfdaspl%2Cmeshpa%7Cmesh%2Cdisease%7Cmedrt%2Cchem%7Cfdaspl%2Cmoa%7Cfdaspl%2Cpe%7Cfdaspl%2Cpk%7Cmedrt%2Ctc%7Cfmtsme%2Cva%7Cva%2Cdispos%7Csnomedct%2Cstruct%7Csnomedct%2Ctherap%7Csnomedct%2Cschedule%7Crxnorm. Accessed March 22, 2023.

[44] Rxclass: opioids. U.S. National Library of Medicine. https://mor.nlm.nih.gov/RxClass/search?query=Opioids+in+combination+with+non-opioid+analgesics%7CATC1-4&searchBy=class&sourceIds=&drugSources=atc1-4%7Catc%2Cepc%7Cfdaspl%2Cmeshpa%7Cmesh%2Cdisease%7Cmedrt%2Cchem%7Cfdaspl%2Cmoa%7Cfdaspl%2Cpe%7Cfdaspl%2Cpk%7Cmedrt%2Ctc%7Cfmtsme%2Cva%7Cva%2Cdispos%7Csnomedct%2Cstruct%7Csnomedct%2Ctherap%7Csnomedct%2Cschedule%7Crxnorm. Accessed March 14, 2023.

[45] Rxclass: opioids in combination with non-opioid analgesics. U.S. National Library of Medicine. https://mor.nlm.nih.gov/RxClass/search?query=OPIOID+ANALGESICS%7CVA&searchBy=class&sourceIds=&drugSources=atc1-4%7Catc%2Cepc%7Cfdaspl%2Cmeshpa%7Cmesh%2Cdisease%7Cmedrt%2Cchem%7Cfdaspl%2Cmoa%7Cfdaspl%2Cpe%7Cfdaspl%2Cpk%7Cmedrt%2Ctc%7Cfmtsme%2Cva%7Cva%2Cdispos%7Csnomedct%2Cstruct%7Csnomedct%2Ctherap%7Csnomedct%2Cschedule%7Crxnorm. Accessed March 14, 2023.

[46] Pullen S, Marconi VC, Del Rio C, Head C, Nimmo M, O’Neil J, From Silos to Solidarity: Case Study of a Patient-Centered, Integrative Approach to Opioid Tapering and Chronic Pain Mitigation in a Multidisciplinary AIDS Clinic. Journal of AIDS and HIV Treatment. 2021;3(1):4–11. 34263265 10.33696/AIDS.3.012 PMC8277158

