# Supplementary material for: Painful to Discuss: The Intersection of Chronic Pain, Mental Health, and Analgesic Use among People with HIV
Source: J AIDS HIV Treat. Author manuscript; Available in PMC 2023 Dec 7. (PMC10703349; doi:10.33696/aids.5.046)
Supplement: JAHT-23-046_Supplementary_File [file NIHMS1944795-supplement-JAHT-23-046_Supplementary_File.zip › Appendix-Appendix_1___Mental_Health.docx]

Appendix 1: ICD-10 Codes – Mental Health

| **Mental health Diagnosis** | **ICD10 code** |
| --- | --- |
| Depression, unspecified depression type | F32.A |
| Recurrent major depressive disorder, remission status unspecified (CMS-HCC) | F33.9 |
| Generalized anxiety disorder | F41.1 |
| Anxiety | F41.9 |
| PTSD (post-traumatic stress disorder) | F43.10 |
| Chronic post-traumatic stress disorder (PTSD) | F43.12 |
| Adjustment disorder with anxious mood | F43.22 |
| History of domestic physical abuse in adult | Z91.410 |
| History of psychological trauma | Z91.49 |
