# Supplementary material for: Painful to Discuss: The Intersection of Chronic Pain, Mental Health, and Analgesic Use among People with HIV
Source: J AIDS HIV Treat. Author manuscript; Available in PMC 2023 Dec 7. (PMC10703349; doi:10.33696/aids.5.046)
Supplement: JAHT-23-046_Supplementary_File [file NIHMS1944795-supplement-JAHT-23-046_Supplementary_File.zip › Appendix-Appendix_2___Pain.docx]

Appendix 2: ICD-10 Codes - Pain

| **Pain Diagnosis** | **ICD10** |
| --- | --- |
| Acute left-sided low back pain without sciatica | 724.2 |
| Other vascular headache | G44.1 |
| Neuropathy of both upper extremities | G56.93 |
| Neuropathic pain of both legs | G57.93 |
| Idiopathic small and large fiber sensory neuropathy | G60.8 |
| Hereditary and idiopathic neuropathy, unspecified | G60.9 |
| Disease related peripheral neuropathy | G62.89 |
| Acquired polyneuropathy | G62.9 |
| Jackson pratt drain site pain | G89.18 |
| Chronic intractable pain | G89.29 |
| Pain around nail | IMO0002 |
| Primary generalized (osteo)arthritis | M15.0 |
| Degenerative joint disease involving multiple joints | M15.9 |
| Patellofemoral pain syndrome of right knee | M22.2X1 |
| Patella-femoral syndrome, left | M22.2X2 |
| Patellofemoral pain syndrome | M22.2X9 |
| Ache in joint | M25.50 |
| Acute pain of right shoulder | M25.511 |
| Acromioclavicular joint pain, left | M25.512 |
| AC joint pain | M25.519 |
| Acute hip pain, right | M25.551 |
| Acute hip pain, left | M25.552 |
| Acute hip pain, unspecified laterality | M25.559 |
| Acute knee pain, right | M25.561 |
| Acute knee pain, left | M25.562 |
| Acute knee pain, unspecified laterality | M25.569 |
| Ankle joint pain, right | M25.571 |
| Acute left ankle pain | M25.572 |
| Acute ankle pain, unspecified laterality | M25.579 |
| Sciatica due to displacement of lumbar intervertebral disc | M51.16 |
| Sciatica associated with disorder of lumbar spine | M53.86 |
| Sciatica associated with disorder of lumbar spine | M53.9 |
| Acute muscle stiffness of neck | M54.2 |
| Pain in lower extremity due to sciatica | M54.30 |
| Back pain with right-sided sciatica | M54.31 |
| Back pain with left-sided sciatica | M54.32 |
| Acute back pain with sciatica | M54.40 |
| Acute back pain with sciatica, right | M54.41 |
| Acute back pain with sciatica, left | M54.42 |
| Acute bilateral low back pain without sciatica | M54.5 |
| Acute bilateral thoracic back pain | M54.6 |
| Pain in deltoid | M79.1 |
| Muscle pain, lumbar | M79.18 |
| Peripheral neuralgia | M79.2 |
| Arm pain, anterior, right | M79.601 |
| Arm pain, anterior, left | M79.602 |
| Arm pain | M79.603 |
| Acute leg pain, right | M79.604 |
| Acute leg pain, left | M79.605 |
| Leg pain | M79.606 |
| Acute extremity pain | M79.609 |
| Pain in right axilla | M79.621 |
| Left axillary pain | M79.622 |
| Axillary pain | M79.629 |
| Pain of right forearm | M79.631 |
| Left forearm pain | M79.632 |
| Forearm pain | M79.639 |
| Hand pain, right | M79.641 |
| Hand pain, left | M79.642 |
| Hand discomfort | M79.643 |
| Finger pain, right | M79.644 |
| Finger pain, left | M79.645 |
| Finger pain | M79.646 |
| Pain in right thigh | M79.651 |
| Left thigh pain | M79.652 |
| Acute thigh pain | M79.659 |
| Calf pain, right | M79.661 |
| Calf pain, left | M79.662 |
| Calf pain | M79.669 |
| Acute foot pain, right | M79.671 |
| Acute foot pain, left | M79.672 |
| Foot arch pain | M79.673 |
| Great toe pain, right | M79.674 |
| Great toe pain, left | M79.675 |
| Great toe pain | M79.676 |
| Pain in left radius | M89.8X3 |
| Aching headache | R51 |
| Aches | R52 |
|  |  |

Appendix 2: ICD-10 Codes – Mental Health
