# Supplementary material for: Painful to Discuss: The Intersection of Chronic Pain, Mental Health, and Analgesic Use among People with HIV
Source: J AIDS HIV Treat. Author manuscript; Available in PMC 2023 Dec 7. (PMC10703349; doi:10.33696/aids.5.046)
Supplement: JAHT-23-046_Supplementary_File [file NIHMS1944795-supplement-JAHT-23-046_Supplementary_File.zip › Appendix-Appendix_3___Analgesics.docx]

Appendix 3 - Analgesics

| MED |
| --- |
| ACETAMINOPHEN |
| ACETAMINOPHEN / CODEINE |
| ACETAMINOPHEN / TRAMADOL |
| ALMOTRIPTAN |
| ALOXIPRIN |
| AMINOPYRINE |
| ANTIPYRINE |
| ASPIRIN |
| ASPIRIN / CODEINE |
| ATOGEPANT |
| BENORILATE |
| BUPRENORPHINE |
| BUTORPHANOL |
| CELECOXIB / TRAMADOL |
| CHOLINE |
| CLONIDINE |
| CODEINE / IBUPROFEN |
| DEXTROMORAMIDE |
| DEZOCINE |
| DIFLUNISAL |
| DIHYDROCODEINE |
| DIHYDROERGOTAMINE |
| DIPYRONE |
| ELETRIPTAN |
| EPTINEZUMAB |
| ERENUMAB |
| ERGOTAMINE |
| ETHENZAMIDE |
| FENTANYL |
| FLOCTAFENINE |
| FLUPIRTINE |
| FONAZINE |
| FREMANEZUMAB |
| FROVATRIPTAN |
| GALCANEZUMAB |
| HYDROMORPHONE |
| IBUPROFEN / OXYCODONE |
| IMIDAZOLE-2-HYDROXYBENZOATE |
| IPRAZOCHROME |
| LASMIDITAN |
| LISURIDE |
| MEPERIDINE |
| MEPTAZINOL |
| METHOXYFLURANE |
| METHYSERGIDE |
| MORPHINE |
| MORPHOLINE SALICYLATE |
| NALBUPHINE |
| NALOXONE / OXYCODONE |
| NALOXONE / TILIDINE |
| NALTREXONE / OXYCODONE |
| NARATRIPTAN |
| NEFOPAM |
| OLICERIDINE |
| OPIUM |
| OXETORONE |
| OXYCODONE |
| OXYMORPHONE |
| PAPAVERETUM |
| PENTAZOCINE |
| PHENACETIN |
| PHENAZOCINE |
| PIRINITRAMIDE |
| PIZOTYLINE |
| PROPOXYPHENE |
| PROPYPHENAZONE |
| RIMEGEPANT |
| RIZATRIPTAN |
| SALICYLAMIDE |
| SALICYLIC ACID |
| SALSALATE |
| SUMATRIPTAN |
| TAPENTADOL |
| TILIDINE |
| TRAMADOL |
| UBROGEPANT |
| ZICONOTIDE |
| ZOLMITRIPTAN |
| 2-DIETHYLAMINOETHANOL |
| ACECLOFENAC |
| ACEMETACIN |
| ACETAMINOPHEN / ASPIRIN / CAFFEINE |
| ACETAMINOPHEN / BUTALBITAL / CAFFEINE |
| ACETAMINOPHEN / HYDROCODONE |
| ACEXAMIC ACID |
| ADAPALENE |
| ADAPALENE / BENZOYL PEROXIDE |
| ALMINOPROFEN |
| ALUMINUM HYDROXIDE / ASPIRIN / MAGNESIUM HYDROXIDE |
| ANTIPYRINE |
| APAZONE |
| APREMILAST |
| ASPIRIN |
| ASPIRIN / MEPROBAMATE |
| BAICALIN |
| BALSALAZIDE |
| BENORILATE |
| BROMFENAC |
| BUFEXAMAC |
| BUMADIZONE |
| BUTIBUFEN |
| CARPROFEN |
| CARYOPHYLLENE |
| CELECOXIB |
| CHLOROQUINE |
| CHOLINE MAGNESIUM TRISALICYLATE |
| CHRYSAROBIN |
| CLONIXIN |
| CURCUMIN |
| DEXKETOPROFEN |
| DICLOFENAC |
| DIFLUNISAL |
| DIPYRONE |
| DROXICAM |
| ECALLANTIDE |
| ETANERCEPT |
| ETHENZAMIDE |
| ETODOLAC |
| ETOFENAMATE |
| ETORICOXIB |
| EVENING PRIMROSE OIL |
| FELBINAC |
| FENBUFEN |
| FENOPROFEN |
| FEPRAZONE |
| FERULATE |
| FLOCTAFENINE |
| FLUNIXIN |
| FLURBIPROFEN |
| GLUCAMETACIN |
| IBUPROFEN |
| ICATIBANT |
| IMIDAZOLE-2-HYDROXYBENZOATE |
| INDOBUFEN |
| INDOMETHACIN |
| KEBUZONE |
| KETOPROFEN |
| KETOROLAC |
| LONAZOLAC |
| LORNOXICAM |
| LOXOPROFEN |
| MAGNESIUM SALICYLATE |
| MAGNOLOL |
| MASOPROCOL |
| MECLOFENAMATE |
| MECLOFENAMIC ACID |
| MEFENAMATE |
| MELOXICAM |
| MESALAMINE |
| MOFEBUTAZONE |
| MOFEZOLAC |
| NABUMETONE |
| NAPROXEN |
| NEPAFENAC |
| NIFLUMIC ACID |
| NIMESULIDE |
| OLOPATADINE |
| OLSALAZINE |
| ORGOTEIN |
| OXAPROZIN |
| OXYPHENBUTAZONE |
| PALMIDROL |
| PARECOXIB |
| PARTHENOLIDE |
| PEONIFLORIN |
| PHENYLBUTAZONE |
| PIMECROLIMUS |
| PIRFENIDONE |
| PIROXICAM |
| PROGLUMETACIN |
| PROPYPHENAZONE |
| ROFECOXIB |
| ROSMARINATE |
| SALICIN |
| SALICYLAMIDE |
| SALICYLIC ACID |
| SALSALATE |
| SERRATIOPEPTIDASE |
| SULFASALAZINE |
| SULINDAC |
| SUPROFEN |
| TENOXICAM |
| TERIFLUNOMIDE |
| TIAPROFENATE |
| TOLFENAMIC ACID |
| TOLMETIN |
| TRANILAST |
| TRIBENOSIDE |
| URSOLATE |
| VALDECOXIB |
| ZILEUTON |
| ZOMEPIRAC |
| ACETAMINOPHEN / CODEINE |
| ACETAMINOPHEN / TRAMADOL |
| ASPIRIN / CODEINE |
| CELECOXIB / TRAMADOL |
| CODEINE / IBUPROFEN |
| IBUPROFEN / OXYCODONE |
| ACETAMINOPHEN / BENZHYDROCODONE |
| ACETAMINOPHEN / BUTALBITAL / CAFFEINE / CODEINE |
| ACETAMINOPHEN / CAFFEINE / DIHYDROCODEINE |
| ACETAMINOPHEN / CODEINE |
| ACETAMINOPHEN / HYDROCODONE |
| ACETAMINOPHEN / OXYCODONE |
| ACETAMINOPHEN / TRAMADOL |
| ALFENTANIL |
| ASPIRIN / BUTALBITAL / CAFFEINE / CODEINE |
| ASPIRIN / CARISOPRODOL / CODEINE |
| ASPIRIN / OXYCODONE HYDROCHLORIDE / OXYCODONE TEREPHTHALATE |
| BELLADONNA ALKALOIDS / OPIUM |
| BUPRENORPHINE |
| BUPRENORPHINE / NALOXONE |
| BUTORPHANOL |
| CELECOXIB / TRAMADOL |
| CODEINE |
| FENTANYL |
| HYDROCODONE |
| HYDROCODONE / IBUPROFEN |
| HYDROMORPHONE |
| IBUPROFEN / OXYCODONE |
| LEVORPHANOL |
| MEPERIDINE |
| METHADONE |
| MORPHINE |
| MORPHINE / NALTREXONE |
| NALBUPHINE |
| NALOXONE / PENTAZOCINE |
| OLICERIDINE |
| OXYCODONE |
| OXYMORPHONE |
| REMIFENTANIL |
| SUFENTANIL |
| TAPENTADOL |
| TRAMADOL |
| ACETAMINOPHEN / BENZHYDROCODONE |
| ACETAMINOPHEN / BUTALBITAL / CAFFEINE / CODEINE |
| ACETAMINOPHEN / CAFFEINE / DIHYDROCODEINE |
| ACETAMINOPHEN / CODEINE |
| ACETAMINOPHEN / HYDROCODONE |
| ACETAMINOPHEN / OXYCODONE |
| ACETAMINOPHEN / TRAMADOL |
| ALFENTANIL |
| ASPIRIN / BUTALBITAL / CAFFEINE / CODEINE |
| ASPIRIN / OXYCODONE |
| ASPIRIN / OXYCODONE HYDROCHLORIDE / OXYCODONE TEREPHTHALATE |
| BUPRENORPHINE |
| BUPRENORPHINE / NALOXONE |
| BUTORPHANOL |
| CELECOXIB / TRAMADOL |
| CODEINE |
| FENTANYL |
| HYDROCODONE |
| HYDROCODONE / IBUPROFEN |
| HYDROMORPHONE |
| MEPERIDINE |
| METHADONE |
| MORPHINE |
| NALBUPHINE |
| OLICERIDINE |
| OXYCODONE |
| OXYMORPHONE |
| REMIFENTANIL |
| SUFENTANIL |
| TAPENTADOL |
| TRAMADOL |
